# Effect of nausea and vomiting during pregnancy on mother-to-infant bonding and the mediation effect of postpartum depression: the Japan Environment and Children’s Study

**DOI:** 10.1186/s12884-023-06014-5

**Published:** 2023-10-02

**Authors:** Gui Yang, Aya Hisada, Midori Yamamoto, Akiko Kawanami, Chisato Mori, Kenichi Sakurai, The Japan Environment, Children’s Study (JECS) Group, Michihiro Kamijima, Shin Yamazaki, Yukihiro Ohya, Reiko Kishi, Nobuo Yaegashi, Koichi Hashimoto, Shuichi Ito, Zentaro Yamagata, Hidekuni Inadera, Takeo Nakayama, Tomotaka Sobue, Masayuki Shima, Seiji Kageyama, Narufumi Suganuma, Shoichi Ohga, Takahiko Katoh

**Affiliations:** 1 Department of Nutrition and Metabolic Medicine, Center for Preventive Medical Sciences, Chiba University, 1-33 Yayoicho, Inageku, Chiba Japan; 2 Department of Sustainable Health Science, Center for Preventive Medical Sciences, Chiba University, 1-33 Yayoicho, Inageku, Chiba Japan; 3 Department of Bioenvironmental Medicine, Graduate School of Medicine, Chiba University, 1-8-1 Inohana, Chuo-Ku, Chiba Japan; 4 Department of Occupational and Environmental Health, Graduate School of Medical Sciences, Nagoya City University, 1 Kawasumi, Mizuho-Cho, Mizuho-Ku, Nagoya, Japan

**Keywords:** Nausea and vomiting during pregnancy, Mother-to-infant bonding, Postpartum depression, Birth cohort

## Abstract

**Background:**

Mother-to-infant bonding (MIB) is critical for the health and well-being of the mother and child. Furthermore, MIB has been shown to boost the social-emotional development of infants, while also giving mothers a sense of happiness in raising their children. Nausea and vomiting during pregnancy (NVP) is a normal complication of pregnancy, occurring in approximately 50–90% of pregnant women in the early stages of pregnancy. Despite widespread knowledge of MIB and postpartum depression, little research attention has been given to the effects of NVP on MIB. This study aimed to investigate the relationship between NVP and MIB and the mediating effects of postpartum depression.

**Methods:**

We analyzed the data of 88,424 infants and 87,658 mothers from the Japan Environment and Children’s Study (JECS), which is a government-funded nationwide birth prospective cohort study. The Japanese version of the Mother-to-Infant Bonding Scale (MIBS-J) was used to assess MIB, and the Edinburgh Postpartum Depression Scale (EPDS) was utilized to assess postpartum depression. We divided participants into four groups according to a self-reported questionnaire assessing NVP (No NVP, Mild NVP, Moderate NVP, and Severe NVP). MIB disorder was defined as a MIBS-J score ≥ 5. Logistic analysis was performed to evaluate the effect of NVP on MIB disorder at one year after delivery. A mediation analysis was conducted to examine whether postpartum depression mediated the association between NVP and MIBS-J scores.

**Results:**

The logistic regression analysis results revealed reduced risks of MIB disorder among mothers with Moderate NVP (adjusted OR 0.93; 95% confidence interval, 0.86–0.99) and Severe NVP (adjusted OR 0.81; 95% confidence interval, 0.74–0.89), compared to those with No NVP. The mediation analysis revealed that NVP positively correlated with MIBS-J score in the indirect effect via postpartum depression, while NVP (Mild NVP, Moderate NVP, and Severe NVP) negatively correlated with MIBS-J score in the direct effect.

**Conclusion:**

The risks of MIB disorder were reduced in the Moderate NVP and Severe NVP mothers, although NVP inhibited the development of MIB via postpartum depression. The development of effective interventions for postpartum depression is important to improve MIB among mothers with NVP.

## Background

Mother-to-infant bonding (MIB) refers to the emotions and feelings that a mother experiences toward her child while caring for her child [1, 2]. These emotions and feelings, such as love, connection, and contentment, are inherited from the mother [2]. MIB is critical for the health and well-being of both the mother and the child. Furthermore, MIB has been shown to boost the social-emotional development of infants, while also giving mothers a sense of happiness in raising their children [3–5]. In contrast, poor MIB has been noted as a risk factor for the growth and development of children, similar to child neglect or abuse [6]. In recent years, studies have reported that a large number of mothers experience MIB disorder [7–11]. In Japan, for instance, MIB disorder reportedly affects 10–21% of mothers [12–14]. Research has also revealed that the mother–child relationship during the first year after delivery is an important predictor of social relationship development in the child’s later life [15]. This is even as several studies have found that the development of mother–child bonding starts during pregnancy and continues until early infancy [1, 16] and that maternal antenatal bonding is positively correlated with MIB after delivery [17–20]. Consequently, it is important to identify the factors associated with mother–child bonding and prescribe measures to prevent MIB disorder as early as possible.

Several factors such as maternal age, education level, household income, marital status, social support [21], number of deliveries [22, 23], feelings about pregnancy [24, 25], maternal personality [26], preterm birth [10, 27], perinatal complication [28, 29], and intimate partner violence [30, 31] have been reported as likely determinants of MIB. Above all, studies have reported that maternal mental status such as psychological distress and postpartum depression [32–37] also affect MIB development. Similarly, the Japan Environment and Children Study (JECS) revealed that mothers experiencing postpartum depression at multiple points postpartum are more likely to have poor MIB [33]. Relatedly, postpartum depressive symptoms at multiple points until four months have been found to be associated with the development of MIB until fourteen months [36]. Furthermore, maternal depressive symptoms are associated with MIB postpartum [17, 35], just as bonding disorders are often present in postpartum depression [37].

Nausea and vomiting during pregnancy (NVP) can affect maternal physical and mental health during pregnancy and the postpartum period [38–40]. NVP is a normal complication of pregnancy, often known as morning sickness, occurring in approximately 50–90% of pregnant women in the early stages of pregnancy [41–44]. Furthermore, approximately 0.3–2% of pregnant women develop hyperemesis gravidarum (HG), a serious type of NVP characterized by weight loss, dehydration, and electrolyte and metabolic abnormalities [45, 46]. However, studies on the relationship between NVP and MIB are limited. A previous study revealed that HG negatively affects maternal antenatal bonding, nevertheless, this negative effect is time-limited to early pregnancy and disappears when HG symptoms disappear [47]. Another study reported that HG is not directly associated with MIB at six weeks after delivery [48]. On the other hand, NVP has been linked to mental health in pregnant women, regardless of the severity of its symptoms [49]. Additionally, women who experienced NVP are more likely to experience postpartum depression [50, 51]. Therefore, we speculate that NVP, including relatively mild cases as a risk factor for postpartum depression, affects the development of MIB. Previous JECS studies suggest that NVP induces physical and mental anguish in mothers, which is positively associated with postpartum depression [50], and postpartum depression inhibits the development of MIB [33]. The findings of these two studies show a predictable relationship between NVP, postpartum depression, and MIB. However, they did not establish how NVP affects MIB via postpartum depression. Therefore, the aim of our study was to investigate the effect of NVP on MIB at one year after delivery. To this end, mediation analysis was used to analyze the direct effects of NVP on MIB and the indirect effects via postpartum depression, by severity of NVP.

## Methods

### Study design

We analyzed the existing dataset of the JECS, which is a nationwide government-funded birth cohort study that started in 2011. The study enrollment period ended in March 2014 [52]. The JECS aims to assess the impact of environmental exposure during the prenatal stage and early childhood on children’s health and development [53, 54]. Pregnant women were recruited from 15 Regional Centers situated throughout Japan.

### Study data

**Fig. 1:**
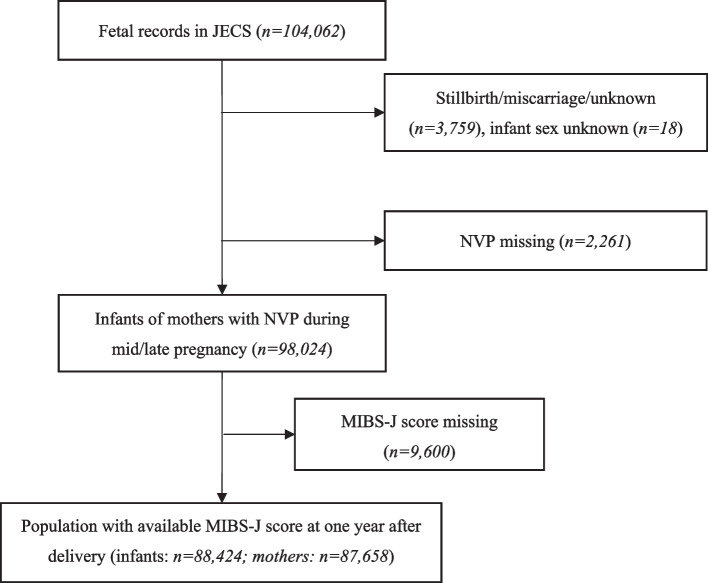
Participant flow diagram. Note: JECS = The Japan Environment and Children’s Study. NVP = nausea and vomiting during pregnancy. MIBS-J score = the Japanese version of the Mother-to-Infant Bonding Scale score

The current study was based on the dataset “jecs-ta-20190930,” which contained 104,062 fetal records. After removing records of miscarriage, stillbirth, and missing birth data (*n* = 3,759), as well as records missing data on the sex of the infants (*n* = 18) and the mothers’ NVP (*n* = 2,261), 98,024 fetal records of infants with mothers with NVP remained. Finally, the study population consisted of 88,424 infants and 87,658 mothers after eliminating missing data on the MIB at one year after delivery (*n* = 9,600) (Fig. 1).

### Measurements

#### Nausea and vomiting during pregnancy (NVP)

We collected information on NVP from self-reported questionnaires administered during mid/late pregnancy. Mothers were asked, “Did you have morning sickness during the first 12 weeks of pregnancy?” (1 = never, 2 = nausea but no vomiting, 3 = vomiting but being able to eat, 4 = vomiting and being unable to eat). We classified participants as No NVP, Mild NVP, Moderate NVP, and Severe NVP, respectively [50, 55].

#### Mother-to-infant bonding (MIB)

MIB was assessed at one year after delivery using the Japanese version of the Mother-to-Infant Bonding Scale (MIBS-J) [56]. The MIBS-J consists of ten items with total scores ranging from 0 to 30. Each item is scored on a four-point scale from 0 to 3. Higher scores indicated worse MIB. We considered MIBS-J scores of five or more as likely to have MIB disorder. This cut-off (4/5) is used in the Japanese version of MIBS [13, 14, 26]. The Cronbach’s alpha of MIBS-J is 0.72 in our study.

#### Postpartum depression

Postpartum depression was assessed at one month after delivery using the Japanese version of the Edinburgh Postnatal Depression Scale (EPDS) [57]. The EPDS consists of ten items with total scores ranging from 0 to 30, each rated on a four-point scale from 0 to 3. A higher score implies more severe depressive symptoms. The EPDS Japanese version's Cronbach's alpha was 0.78, indicating potent internal consistency.

### Covariates

We picked the following covariates to adjust the logistic regression and mediation models, based on other studies on the risk factors of NVP and MIB [19, 58]: maternal age, multiple births, parity, maternal educational level, paternal educational level, annual household income, marital status, feelings about the pregnancy, psychological distress (assessed by the Kessler Psychological Distress Scale [K6] during mid/late pregnancy) [59], verbal abuse from a partner, physical abuse from a partner, maternal alcohol intake, maternal smoking status, paternal smoking status, and infant’s sex. The categorization of these variables is shown in Table 1.

**Table 1:** Demographic and obstetric characteristics of mothers (*n* = 87,658) and infants (*n* = 88,424)

Characteristics	Mean/*n*	SD/%
EPDS score at 1 month after birth^a^	5.8	2.4
NVP status, *n* (%)^b^		
No NVP	15,038	17.2
Mild NVP	37,778	43.1
Moderate NVP	25,276	28.8
Severe NVP	9,566	10.9
Maternal age, years, *n* (%)^b^		
≤ 29	31,806	36.3
≥ 30	55,848	63.7
Marital status, *n* (%)^b^		
Married	83,486	95.2
Single/Divorced/widowed	3,491	3.7
Feelings about the pregnancy, *n* (%)^b^		
Delighted	79,398	90.6
Negative feelings	7,595	8.7
Parity, *n* (%) ^b^		
Primipara	35,164	40.1
Multipara	50,425	57.5
K6 score during mid/late pregnancy, *n* (%)^b^		
≤ 4	62,907	71.8
≥ 5	24,650	28.1
Maternal educational level, years, *n* (%)^b^		
≤ 12	30,451	34.7
> 12	56,907	64.9
Paternal educational level, years, *n* (%)^b^		
≤ 12	37,326	42.6
> 12	49,545	56.5
Maternal smoking status, *n* (%)^b^		
Never a smoker	51,388	58.6
Ex-smoker who quit before pregnancy	20,934	23.9
Ex-smoker who quit during pregnancy	11,259	12.8
Current smoker	3,446	3.9
Paternal smoking status, *n* (%)^b^		
Never a smoker	24,155	27.6
Ex-smoker who quit before pregnancy	20,433	23.3
Ex-smoker who quit during pregnancy	2,500	2.9
Current smoker	39,221	44.7
Maternal alcohol intake, *n* (%)^b^		
Never drank	29,329	33.5
Ex-drinker who quit before pregnancy	14,835	16.9
Current drinker	42,837	48.9
Annual household income, *n* (%)^b^		
< 4 million, Yen	32,249	36.79
4–8 million, Yen	40,648	46.4
> 8 million, Yen	5,460	6.2
Verbal abuse from a partner, *n* (%)^b^		
No	75,734	86.4
Yes	11,591	13.2
Physical abuse from a partner, *n* (%)^b^		
No	86,447	98.6
Yes	988	1.1
Mother-to-Infant Bonding score at 1 year after birth^c^	1.95	2.3
Mother-to-Infant Bonding score at 1 year after birth^c^		
≤ 4	78,219	88.5
≥ 5	10,205	11.5
Multiple births, *n* (%)^c^		
No	86,832	98.2
Yes	1,592	1.8
Infant’s sex, *n* (%)^c^		
Male	45,335	51.3
Female	43,089	48.7

*SD* Standard deviation, *EPDS score* Edinburgh Postpartum Depression Scale score, *NVP* nausea and vomiting during pregnancy, *K6 score* psychological distress (the Kessler Psychological Distress Scale score)

^a^Descriptive statistics based on mothers with EPDS data (*n* = 86,064)

^b^Descriptive statistics based on mothers (*n* = 87,658)

^c^Descriptive statistics based on infants (*n* = 88,424)

### Statistical analysis

**Fig. 2:**
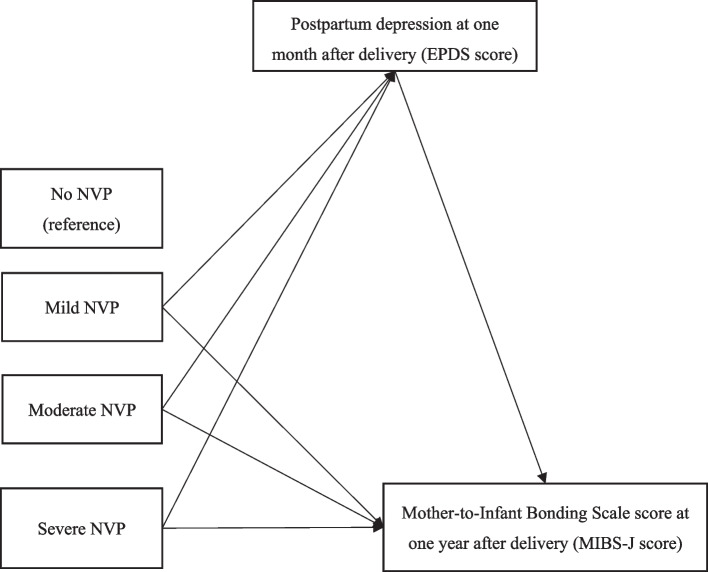
Mediating effect of EPDS score on the association between NVP and MIBS-J score – Mediation model. NVP = nausea and vomiting during pregnancy; EPDS score = Edinburgh Postnatal Depression Scale score; MIBS-J score = the Japanese version of the Mother-to-Infant Bonding Scale score

The demographic characteristics of the participants were presented using descriptive statistics. All continuous variables included in this study, such as the total scores for the EPDS and MIBS-J, were calculated to determine means and standard deviations. Logistic analysis was performed to evaluate the relationship between NVP and MIB disorder at one year after delivery, with NVP (4 categories) as the independent variable (reference, No NVP) and the MIBS-J binary variable (MIB disorder, ≥ 5) as the dependent variable. Odds ratios (ORs) and 95% confidence intervals (CIs) were determined in crude (cOR) and adjusted (aOR) models controlling for covariates. A mediation analysis was conducted to examine whether postpartum depression mediated the association between NVP and MIBS-J scores. In the mediation model (Fig. 2), NVP (four categories) was used as the independent variable, with No NVP as the reference group. The continuous MIBS-J score was used as the dependent variable, and the EPDS score was used as the mediator variable. The relative indirect effect was used to determine whether the independent variable influenced the dependent variable through the mediator. Bias-corrected (BC) bootstrap 95% CIs were obtained for the potential mediators. These were utilized to examine the significance of the relative total and indirect effects, based on 5,000 bootstrap samples. The indirect effect was deemed significant if the BC bootstrap 95% CI did not contain zero.

All statistical analyses were performed using SPSS version 25 software (IBM Corp., Armonk, NY, USA). The mediation analysis was conducted using PROCESS macro for SPSS Ver.4.0 (Andrew F. Hayes).

## Results

Table 1 displays the demographic and obstetric characteristics of the mothers and infants. Of the 88,424 mothers, 78,219 (88.5%) did not report MIB disorder, while 10,205 (11.5%) did. Of the 87,658 mothers, 15,038 (17.2%) were No NVP mothers, 37,778 (43.1%) were Mild NVP mothers, 25,276 (28.8%) were Moderate NVP mothers, and 9,566 (10.9%) were Severe NVP mothers. Regarding maternal age during pregnancy, 71.7% of the mothers were 20 to 34 years old. In addition, 95.2% of the mothers were married, and 90.6% felt happy when they found out they were pregnant.

Table 2 shows the association between NVP and MIB disorder at one year after delivery. We found that mothers with Moderate NVP had a reduced risk of MIB disorder in the adjusted model (aOR 0.83; 95% CI, 0.86, 0.99). Moreover, mothers with Severe NVP had a reduced risk of MIB disorder in the crude model (cOR 0.91; 95% CI, 0.84, 0.98) and the adjusted model (aOR 0.81; 95% CI, 0.74, 0.99). For the Mild NVP mothers, there were no significant differences in either the crude or adjusted models.

**Table 2:** The odds ratio of MIB disorder in relation to the NVP status

NVP status	Crude model	Adjusted model
	cOR (95% CI)	aOR (95% CI)
No NVP	1 (reference)	1 (reference)
Mild NVP	0.97 (0.92, 1.03)	0.96 (0.90, 1.03)
Moderate NVP	0.96 (0.90, 1.03)	0.93 (0.86, 0.99)
Severe NVP	0.91 (0.84, 0.98)	0.81 (0.74, 0.89)

The model was adjusted for maternal age, marital status, feelings about the pregnancy, parity, multiple births, psychological distress (the Kessler Psychological Distress Scale (K6)), maternal and paternal educational level, maternal and paternal smoking, maternal alcohol intake, annual household income, partner’s verbal or physical abuse, and infant’s sex

*MIB* Mother-to-infant bonding, *MIBS-J* Mother-to-Infant Bonding Scale, *NVP* nausea and vomiting during pregnancy, *cOR* crude odds ratio, *aOR* adjusted odds ratio, *CI* confidence interval, MIB disorder defined as MIBS-J score ≥ 5

Table 3 shows the mediating effects of postpartum depression on the association between NVP and MIBS-J score at one year after delivery, in the crude and adjusted models. In the relative indirect effects via postpartum depression, the Mild NVP (standardized path coefficient [β]: 0.022; BC 95% CI: 0.016, 0.027 in the adjusted model), Moderate NVP (β: 0.020; BC 95% CI: 0.015, 0.026), and Severe NVP (β: 0.028; BC 95% CI: 0.020, 0.035) showed positive associations with MIBS-J score (i.e., bonding suppression) compared with the No NVP. In the relative direct effects, Moderate NVP (β: -0.023; 95% CI: -0.101, -0.006) and Severe NVP (β: -0.091; 95% CI: -0.268, -0.147) were negatively associated with MIBS-J scores (i.e., bonding promotion). As the relative total effects, Severe NVP (β: -0.063; 95% CI: -0.207, -0.081) was negatively associated with MIBS-J score (i.e., bonding promotion).

**Table 3:** Mediation effect of NVP on MIBS-J score through EPDS score in mediation analysis

**Relative total effects (NVP → MIBS-J score)**		
	Crude model β (95%CI)	Adjusted model β (95%CI)
No NVP	0 (reference)	0 (reference)
Mild NVP	0.013 (-0.015, 0.073)	0.014 (-0.014, 0.079)
Moderate NVP	0.002 (-0.042, 0.051)	-0.003 (-0.057, 0.042)
Severe NVP	-0.025 (-0.116, 0.002)	-0.063 (-0.207, -0.081)
**Relative direct effects (NVP → MIBS-J score)**		
	Crude model β (95%CI)	Adjusted model β (95%CI)
No NVP	0 (reference)	0 (reference)
Mild NVP	-0.017 (-0.081, -0.001)	-0.008 (-0.062, 0.027)
Moderate NVP	-0.031 (-0.116, -0.028)	-0.023 (-0.101, -0.006)
Severe NVP	-0.083 (-0.246, -0.134)	-0.091 (-0.268, -0.147)
**Relative indirect effects (NVP → EPDS score → MIBS-J score)**		
	Crude model β (BC 95%CI)	Adjusted model β (BC 95%CI)
No NVP	0 (reference)	0 (reference)
Mild NVP	0.030 (0.024, 0.036)	0.022 (0.016, 0.027)
Moderate NVP	0.033 (0.027, 0.040)	0.020 (0.015, 0.026)
Severe NVP	0.058 (0.049, 0.067)	0.028 (0.020, 0.035)

The model was adjusted for maternal age, marital status, feelings about the pregnancy, parity, multiple births, psychological distress (the Kessler Psychological Distress Scale (K6)), maternal and paternal educational level, maternal and paternal smoking, maternal alcohol intake, annual household income, partner’s verbal or physical abuse, and infant’s sex

*NVP* nausea and vomiting during pregnancy, *MIBS-J score* the Japanese version of the Mother-to-Infant Bonding Scale score at one year after delivery, *EPDS score* Edinburgh Postnatal Depression Scale score, *β* standardized path coefficient, *95% CI* 95% confidence interval, *BC 95% CI* bias-corrected bootstrap 95% confidence interval

## Discussion

In our study, 11.5% of mothers developed MIB disorder, which is consistent with other studies that a significant number of mothers experienced MIB disorders after birth [12–14]. Also, we found that 82.8% of pregnant women experienced NVP in the current pregnancy, which is also consistent with other studies that revealed 50–90% of pregnant women experience NVP [41–44]. We found that 10.9% of Japanese mothers had Severe NVP, defined as vomiting and not being able to eat. The percentage was consistent with a birth cohort study in the United Kingdom reporting that 11% of pregnant women experienced Severe NVP [55].

The findings of the logistic regression analysis demonstrate that mothers with Moderate NVP and Severe NVP had a reduced risk of MIB disorder at one year after delivery in our study. Similarly, the total effect of mediation analysis also revealed a negative association with MIBS-J score (i.e., bonding promotion) in the Severe NVP mothers. Mitchell-Jones et al. examined the association between HG and MIB at six weeks after delivery, in which no significant difference was observed [48]. This mismatch between studies may be due to factors including differences in the definition of NVP (questionnaire-based vs. diagnosed), in the timing of MIB evaluation (1 year vs. 6 weeks after delivery), or in ethnicity and dietary patterns.

The findings of our study demonstrate that NVP may have a direct effect on MIB. MIB may be affected by pregnancy-related hormonal changes. It has been found that mothers with a high estrogen/progesterone ratio during pregnancy have more positive attitudes toward their newborns immediately after birth [60]. Although the exact mechanism is unknown, estradiol is thought to stimulate parental behavior by acting on the medial preoptic area of the forebrain and other brain regions related to maternal behavior [61]. Furthermore, estrogen levels have been reported to be higher in pregnant women with NVP than in those without NVP [58, 62]. Therefore, increased estrogen levels during pregnancy may have had a beneficial effect on the development of MIB.

Contrary to the direct effect, the indirect effect via postpartum depression from NVP (Mild NVP, Moderate NVP, and Severe NVP) was found to inhibit the development of MIB at one year after delivery. NVP has also been found to be positively associated with postpartum depression [39, 51], and, postpartum depression inhibits the development of MIB [21]. Although Mitchell-Jones et al. reported the association between HG and postpartum depression, there was no direct association of HG with infant bonding in their study [48]. This result suggests that NVP or HG plays a role in the establishment of infant bonding in a different way from postpartum depression. Our findings highlight that NVP promotes the development of MIB but suppresses it through postpartum depression. Thus, we suggest monitoring the NVP symptoms during pregnancy to provide timely and effective intervention. We believe that if a pregnant woman is cared for from the onset of NVP to reduce the risk of postpartum depression, the risk of poor MIB may also be reduced.

Our study has several strengths. First, it is groundbreaking, as it is, to the best of our knowledge, the first study to examine the association between NVP and MIB, along with the mediation effect of postpartum depression on the association between NVP and MIB. Second, we analyzed data from a large Japanese birth cohort, with the individual characteristics of mothers and children roughly corresponding to Japanese national statistics [54]; so our findings are highly representative of the Japanese population. Despite these clear strengths, our study has some limitations. First, information on NVP was collected using a self-reported questionnaire, while this can reduce the burden on participating mothers to answer the questionnaire, it could also limit the objectivity of the data and could cause recall bias. Second, despite our large sample size, there is still a possibility of coincidental results in the statistical analysis process. Third, even though the results of this study were obtained after adjusting for maternal and paternal educational levels, annual household income, and other confounding factors, the analysis results could be impacted by the change in Japan's current socio-economic situation given that the data was collected from 2011 to 2014. Finally, our study only examined the effect of NVP on MIB at one year after delivery; however, MIB changes over time. Therefore, more research on time-series changes in MIB caused by NVP is required to investigate how the effect of NVP on MIB varies from pregnancy up to one year after delivery.

## Conclusion

Our study revealed that mothers who experienced Moderate NVP and Severe NVP had a lower risk of MIB disorder. In addition, NVP inhibits the development of MIB through postpartum depression. Importantly, our findings suggest that it is essential to provide interventional care for mothers from the onset of NVP to prevent postpartum depression to exert the promoting effect of NVP on MIB. Also, our study provides new insights into the facilitating effect of NVP on MIB when assessing the relationship between NVP and MIB. Future research should investigate the indirect impacts of familial or social support generated by the NVP on MIB. We believe that promoting MIB through intervention benefits both the mental health of the mother and the growth and development of the child.

## Data Availability

Data are unsuitable for public deposition due to ethical restrictions and legal framework of Japan. It is prohibited by the Act on the Protection of Personal Information (Act No. 57 of 30 May 2003, amended on 9 September 2015) to publicly deposit data containing personal information. Ethical Guidelines for Medical and Health Research Involving Human Subjects, enforced by the Japan Ministry of Education, Culture, Sports, Science and Technology and the Ministry of Health, Labour and Welfare, also restricts the open sharing of epidemiologic data. Any questions about data access should be directed to Dr. Shoji F. Nakayama, JECS Program Office, National Institute for Environmental Studies, at jecs-en@nies.go.jp.

## Abbreviations

EPDS: Edinburgh Postpartum Depression Scale
HG: Hyperemesis gravidarum
JECS: Japan Environment and Children’s Study
MIB: Mother-to-infant bonding
MIBS-J: Mother-to-Infant Bonding Scale (Japanese version)
NVP: Nausea and vomiting during pregnancy
